# A Comparison of Human Tracking Systems on a Mobile Robotic Platform

**DOI:** 10.3390/s25196172

**Published:** 2025-10-05

**Authors:** Vlad-Andrei Fara, Sebastian-Ioan Petruc, Razvan Bogdan, Marius Marcu

**Affiliations:** Department of Automation and Computing, Politehnica University Timisoara, 300006 Timisoara, Romania; vlad.fara@student.upt.ro (V.-A.F.); sebastian.petruc@upt.ro (S.-I.P.); marius.marcu@cs.upt.ro (M.M.)

**Keywords:** following robot, tracking system, computer vision

## Abstract

The field of IoT has a growing interest in robotics, and one of the big fields of this interest is human–robot collaboration. A sub-category of the human–robot collaboration is how a robot can follow a human. There are a few methods of following a person; one would be using image recognition, and another way could be using a rope attached to that person. Image recognition is compared to following a person with a rope to find out the benefits and weaknesses of both methods, comparing smoothness, responsiveness, and accuracy. With the comparison of these two systems, the findings presented in this study indicate that the overall responsiveness of the spool-based system is higher than the camera-based system, the directional responsiveness of the camera-based system is higher than the spool-based system, and the distance estimation of the camera-based system is noisier and less reliable than the spool-based system.

## 1. Introduction

There is a real interest in robotics recently, especially in the field of IoT. Mainstream robot vacuums recently gained robotic arms to move items or even became platforms for entire robotic systems; robotic deliveries have also gained traction. This means there is a genuine reason to compare and find out what would be the best solution to human tracking for robots that must follow people around. This could help with both industrial solutions, where a robot should be able to follow a worker to a station, or even personal robots that could help people with carrying heavy items for fatigue alleviation or increased carrying capacity. A robotic platform that can follow its user around can be a great addition in many fields where it could be adapted and extended to accomplish more specialized tasks; for example, a robot that can sow seeds could be utilized as a more cost-effective solution on smaller fields, and it could be reconfigured to have a cargo hold when picking up crops, but this would only be effective for a person to use if it could autonomously follow the person.

Another example would be carrying equipment for an on-site job. A professional could want to haul a large amount of equipment and tools for a more complex job, and it would be beneficial to have an autonomous robot if he must carry out that job by himself. A direct use of these tracking systems in IoT would be to collect environmental data in an area or even establish a network of these tracking systems that follow their owners to obtain real-time data from different areas. The two systems that are the focus of this research are spool-based tracking, which is attached to the person through a rope whose extension and direction are measured and image processing-based tracking, which is camera-based. The camera-based system also gives distance and direction estimations based on the skeleton estimation of the person it is tracking. To study the human tracking systems, they needed to be implemented and compared on a single platform. With that goal in mind, a mobile robotic platform has been implemented, and both systems are able to operate simultaneously to collect data and can be switched from one to the other at any point when in operation.

For safety and ease of use, all the states and some inputs are received by the robotic platform from a remote control, specifically the RadioMaster TX16s Mk. II. (manufactured by Radiomaster, Shenzhen, China) This allows for manual operation when needed, arming and disarming the robotic platform if tracking is lost, switching between modes of operation, and other commands. The experimental data also needs to be grounded in reality and there needs to be a way to visualize the path driven while the experiment was taking place; to that end, all the motor encoders and the servo commands were logged and interpreted to generate the physical path representation. For validating the experimental data, a methodological aspect is recording video of the experiments from both the perspective of the robotic platform and the perspective of the subject; this ensures data collection was done right.

In the context of robotic following systems, various methods were considered, such as infrared beacons, radio-based localization and computer vision. Each of them provides different advantages at the cost of system-wise trade-offs in either cost, accuracy or robustness. This paper focuses primarily on Spool and vision-based methods due to their wide-spread use in the field and their relevance to robots’ mobility. Moreover, IoT technologies are increasingly becoming part of robotic tracking systems, with various opportunities arising for increased connectivity and data sharing for a better integration of mobile systems in real-world scenarios with risks of noise, dynamic pose changes and occlusions.

It is also imperative for human tracking robotic systems to adhere to specific social norms when interacting. Understanding human behavior, such as the differences in the following distance, the approach angle and the responsiveness of the system to fast changes in the movement of the users, directly influences trust and safety; while this paper primarily focuses on the engineering comparisons between camera- and Spool-based systems, it also discusses the strengths and possible limitations of these systems in the human–robot interaction experience. Our study frames comparison through both technical and behavioral perspectives, highlighting the way tracking robotic systems influences both technical performance and social acceptability.

This paper’s contributions can be summarized as a system-wise comparison between camera-based, Spool-based and fusion tracking methods all in one single mobile platform (the vast majority of platforms containing only one human-tracking technology) and the interpretation of results from a human–robot interaction perspective.

## 2. Related Work

Two systems were studied, specifically tethered tracking and image recognition-based following. These would be two tiers of tracking, one that is attached to the person and the other fully internal. All these methods should have their preferred environment; as an example, in crowded areas, image recognition might fail, but using tethered tracking, the platform could follow a person easily; conversely, in a solo trip, the image recognition might prove more convenient. There are strong points and weak points to each of these technologies, and research into this topic could prove valuable. Finally, tether tracking is also mentioned in [1,2].

It comprises a cable spool attached to an encoder, which tells the robot how far the line was extended, and a potentiometer that tracks the horizontal angle at which the tether is tensioned. With these two pieces of information, a robot should not only be able to track a person in real time and extremely accurately but also build a path on which that person walked so that the robot could emulate that path and not take shortcuts so that it does not hit anything in the way. This could work in the manner that origin vectors work, with the robot being the origin and the tether being the vector, and with changes over time, it could add to the list of tether vectors; that way, another set of vectors can be established, which would represent the path the person took, and at that point, the robot would follow the actual path the person took. This approach could solve the problem presented in paper [2], where they switch movement algorithms based on how far from the center the angle points. Although this poses a challenge when applying it on-the-go because of the moving coordinate system established with the origin in the robot, it could be a really good following system.

Another system that was present in the literature is the infrared beacon, which is more prevalent instead, being mentioned in papers such as [3,4], and the reason not to try out localization using Radio can be seen, as attempted in paper [5], where the latency in distance information is significant and the tracking is choppy; instead, focusing on light can eliminate radio transmission and processing latency, reducing the tracking system to the robot only and the beacon as a sort of flashlight only. The infrared beacon following system works by tracking the direction of an infrared beacon blinking at a set frequency, usually at 38 kHz, by using multiple receivers placed in a circular pattern like in paper [3], for omni-directional sensing, while paper [4] only uses IR for alignment. By measuring which receiver senses the signal at what intensity, a directional vector can be constructed that represents the direction of the IR signal. By using two such systems, one could calculate two angles and determine the distance to the beacon at the intersection of said calculated angles, like stereo cameras, which can determine depth. The spacing between two such systems would need to be determined to see how granular the distance measurement can be; ideally, the receivers would not need to be spaced apart more than the width of the robot, but this requires both performing calculations to determine how granular the angle sensing can be and experimental evidence that the determined distance between receivers is sufficient. Based on the accuracy and how much the values vary, it might be possible to implement the path-following approach as described before.

Another system is based on image recognition. There is previous research performed for mobile robotic platforms that use image recognition to track humans, such as [6,7], that can recognize humans and can even estimate the skeleton of the person they are following, like in this paper [8]. The proposed system can infer in what direction and at what distance a person stands or walks and would be a good candidate for research and experimentation in a comparative study. The advantage of such a system is that it does not use any external means, such as a tether attached to the person, and not even a small device like a beacon, such as the IR beacon tracking detailed before. It only uses image information to determine its next steps; when the tracked person goes too far, it will start to follow; if the person starts going to the side, it will start to turn, etc. Due to the uncertain and possibly jittery tracking of the person, it might not be possible to implement a path-tracking algorithm such as the one described for the tether, where just a single bigger spike would send the robot on a zig-zag trajectory. A deeper dive into estimating the skeleton of the person, also called pose estimation, is performed in paper [9], where a comparison between pose estimation models is made. The candidates in this paper are Detectron2, Mediapipe and YOLOv7, among others. Since this paper was published, there have been significant advancements in YOLO models, and it reached version YOLOv11, as illustrated in paper [10], where it is utilized for fall detection based on pose estimation.

The robotic platform is simple but has the feature of carrying a payload to simulate a real mission; for example, it can aid in carrying heavy weights for a person that is prescribed to put the least amount of pressure on their joints as possible. To determine ideal conditions for both the comfort of a person and performance of the robot, the core algorithms for following a tracked person and potentially alternative positions of tracking, there are some research articles such as [8,11,12,13], where these topics are explored more in-depth. An aid in implementing and researching the image recognition system would be described in paper [8], where the authors describe in detail the tracking of the human skeleton using image recognition and establishing an optimal distance of the robot from the person using the skeletal data. Another important piece of information can be extracted from the paper [11], where the researchers determine a set of comfortable distances between a person and the robot. This is important, since a robot following too close could injure a person, and if it follows too far away, it could get in the way of other people that occupy the same area as the human–robot pair.

There are also potential alternative positions of the robot following a human, as described in papers [12,13]; for example, following from the side of the person or in front of the person. This could prove advantageous in certain scenarios but could also pose challenges in setting up the positions and perhaps even motorization of the sensors to be able to track in certain directions; while paper [12] only elaborates on the side-by-side tracking with some experiments, paper [13] instead takes a more general approach to the following algorithm and presents paths taken by following a person behind but also presents a complete following algorithm. This could be helpful in designing the path-following algorithm, instead of directly following the person.

An important system for the robotic platform is steering and throttle control. Since the size and weight are considerable and the wheels grip the terrain significantly, it needs a movement system where wheels will not slip. Performing this also increases the efficiency of the motors. To that end, Ackermann steering is a great model to implement, as seen in papers [14,15,16]. Starting with two-wheel steering in papers [15,16], it can be seen how the steering center lies along the axis that passes through the centers of the rear wheels. In the case of both papers, it is in the back, to take full advantage of the wheels when steering. Paper [15] describes a more physics-based calculation of the Ackermann Steering Model, where every force is considered, but taking advantage of simplifications, paper [16] describes a more mathematical approach to the model, which is useful for the implementation. Paper [14] describes the implementation of the Ackermann steering model on a six-wheeled rover. Simplifying the six-wheeled rover to a four-wheel steering vehicle can be performed by only calculating the four corner wheels of the rover in the paper. Using multiple concentric circles for the wheels to go on for an optimal steering path, requires all the wheels to point to a specific point on the ground. In this case, the center of the rover on the ground plane was utilized to draw the axis perpendicular to the forward movement, on which the steering center lies. Making use of this, the two wheels in a group have the opposite angles when taking the straight orientation as the origin, with each direction, left or right, having different signs. This also creates the need for the two pairs of wheels that sit on two different concentric circles to have different speeds; the outer wheels have greater speed than the average speed of the robotic platform, and the inner wheels have less speed than the average. By using the wheels to steer this way, the turning radius of the rover can be reduced significantly. For safety, ease in unplanned movements, and controlling the states, a remote control is needed to send data to the robotic platform; a good protocol was found in papers [17,18]. ExpressLRS is a fast and reliable open-source protocol [19] utilized primarily for remote-controlled vehicles, typically drones, fixed-wing airplanes, and cars.

ExpressLRS is utilized on a custom wearable remote controller in paper [17] and on a custom but well-packaged hand-held remote controller in paper [18] to control an Unmanned Aerial Vehicle each. This makes it a good choice for the robotic platform, since it needs a remote control with good performance and range, which both papers prove to be adequate since they are utilized in highly dynamic scenarios. In order to be able to compare the two systems accurately, the robotic platform needs to be able to know its precise relative position during an experiment; for that, the DC motor encoders can be utilized alongside the servo motor angles as described in paper [20]. Plotting the path can be achieved by tracking the encoder positions and servo angles at certain timestamps and building the plot point-by-point according to how the platform moved between timestamps. Another paper that evaluates this positioning method and compares it to others is paper [21], where encoder position is compared to ultrasonic distance sensors and Wi-Fi signal strength, with encoder position being the most accurate and consistent.

## 3. Materials and Methods

**Figure 1:**
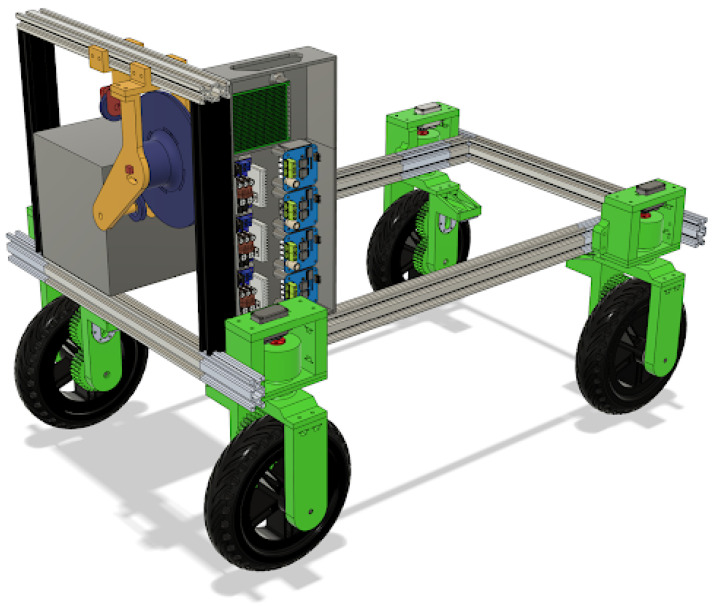
CAD render of the robotic platform.

With the purpose of combining both ways of tracking a person, a robotic platform was projected as being controllable by both systems to compare the results (Figure 1). The platform comprises a chassis on which four independent wheel assemblies, which have both steering and powered wheels, are mounted. These wheels and steering assemblies are all controlled and read by an ESP32 that manages them. To send power to the wheels, DC motor drivers were utilized.

The platform has a robust power distribution system. An LiFePO4 battery is utilized for power, which gets utilized on 3 buses of 5 v, 6–7 v and 12 v. Each of these buses is responsible for a part of the system. The 5 v bus is utilized to power the controllers, the 6–7 v one is utilized for the servos, and the 12 v one is utilized for the DC motors of the wheels. On the topic of power, there was a need to use many Logic Level Converters so that the signals that the controllers are receiving and sending are at the correct voltage. The ESP32 boards use a 3.3 v logic level and are not 5 v tolerant. This is utilized for both reliability and safety concerns.

**Figure 2:**
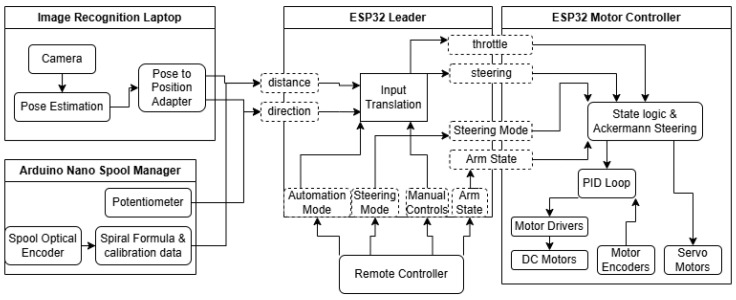
Diagram of data flow and state flow.

The diagram in Figure 2 shows an overview of the system itself, how components interact, and how the states of the robot are passed around so it can accomplish its goals. It can also be seen how the remote control has the final say in everything, so Manual Control can take over at any time necessary. The remote control is the RadioMaster TX16s Mk II. It runs the EdgeTX operating system and has an internal ExpressLRS transmitter, which can also receive telemetry; that way, it knows if the robot is connected or not.

### 3.1. Mechanical Design

The topic of mechanical design is an important one since the platform is of vital importance to test out the results of the ways in which a person can be followed. Some big considerations were price, availability of parts, stability, reliability and upgradability. Some mechanical parameters of this solution are a carrying capacity of 40 kg and a top speed around a light jog, around 8 km/h. To this end, all the load-bearing parts were designed with more internal reinforcement, denser infill and special bearings for the load-bearing axles of the direction assembly of wheels.

**Figure 3:**
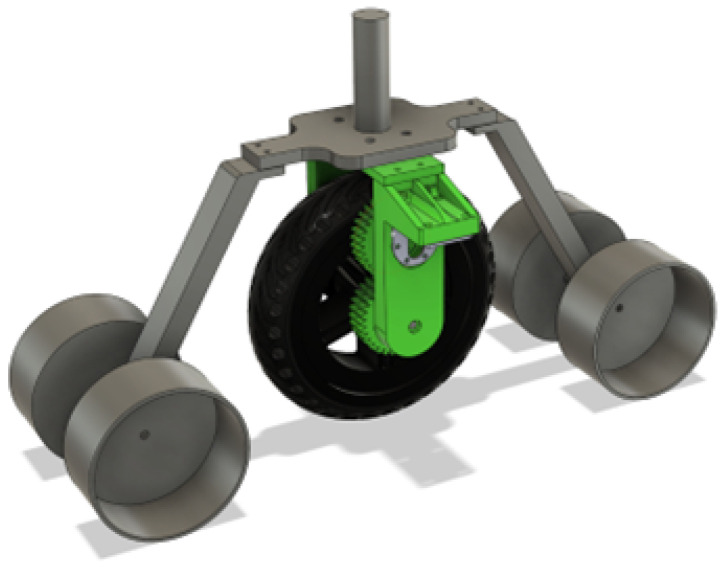
CAD render of DC motor weight test bed.

To test the claim of being able to carry 40 Kg, the test bed for the DC motors in Figure 3 was utilized and loaded with more than 10 KG in weight plates on the shaft of the test bed.

#### 3.1.1. Structure

The robotic platform is assembled around a frame built from X-shaped aluminum extrusions, which can use T-slot nuts and corner connectors. These reside in the slots of the extrusions and have good mechanical grip. The frame is designed to have a cargo area. The cargo area can hold a Euro Box of 60 cm by 40 cm footprint, or two 40 cm by 30 cm boxes side-by-side. The boxes can also be stacked on top of each other within the theoretical limit of 40 kg.

The placement of the tracking systems was a big consideration in creating a raised frontal area. It was designed to be the height of the 60 × 40 Euro Box so that it is streamlined and of a determined height.

**Figure 4:**
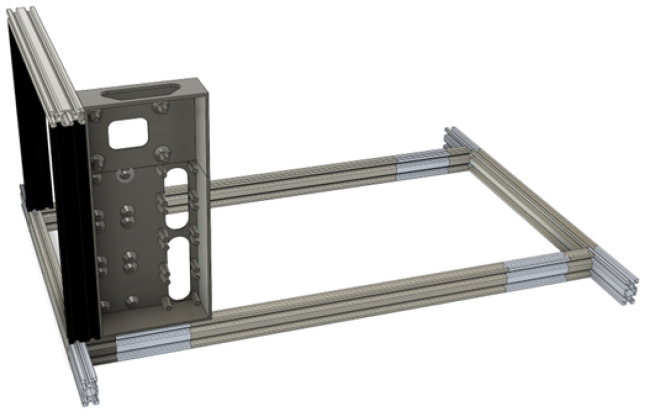
Electronics mounting box.

For custom mounting of electronics and holding the battery in place, custom 3D-printed parts were utilized, presented in Figure 4. There are the parts that hold up the battery in a precise position and a few custom cases that house and lay out the main electronics.

#### 3.1.2. Locomotion

**Figure 5:**
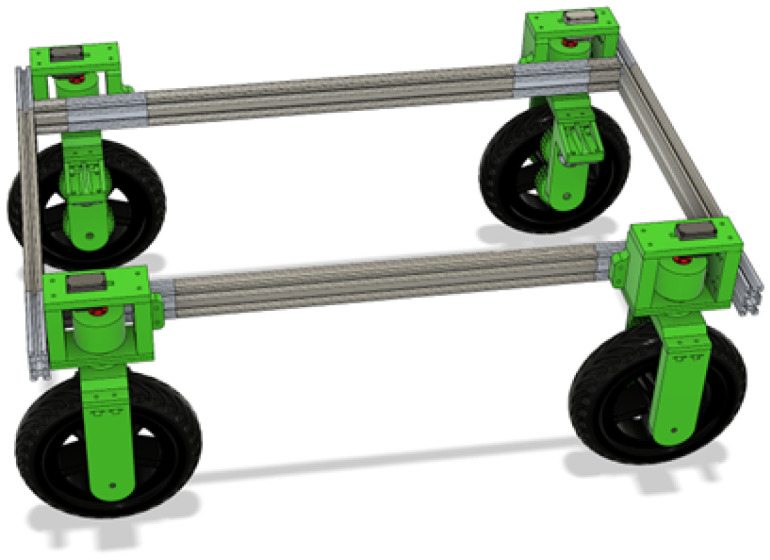
Wheel assemblies mounted to frame.

For moving around, a 3D-printed wheel assembly was designed that got utilized in all four corners. By only rotating and mirroring some of the 3D printed parts, the wheel assembly could be utilized on all four corners of the frame, as seen in Figure 5, thus having a unified design that is easy to modify. There was a lot of symmetry utilized, so that the mechanisms align and balance well.

At first, there was the problem of powering the wheels. The wheels are electric scooter wheels that have a disk brake mounting pattern for which a gear was designed to go over it. The reason for needing a gear was the fact that the wheel axles are not fixed but on bearings. The additional benefit of moving the motor is that it can clear bigger obstacles.

**Figure 6:**
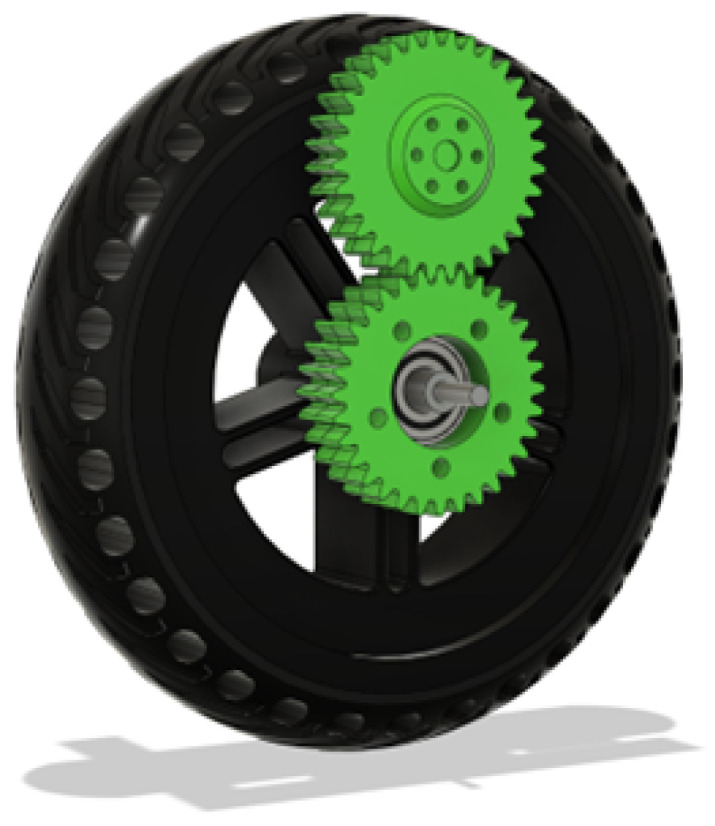
Wheel gear assembly.

Then a matching gear was designed that can mesh into it that is attached to a flange that is on the shaft of the DC motor. It has space to accommodate the shaft if it protrudes too much and 6 m^3^ screw holes that screw into the motor flange. This can be seen in Figure 6.

**Figure 7:**
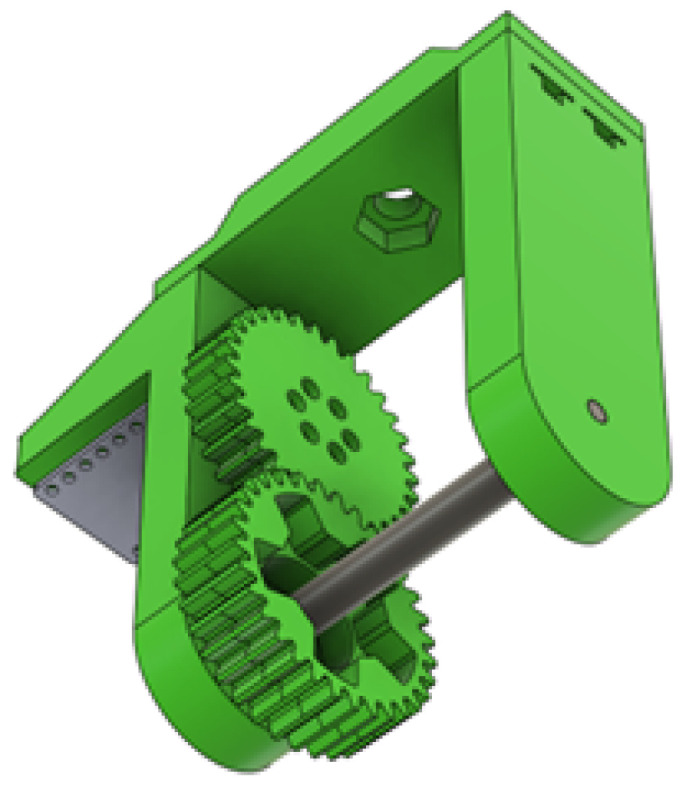
Wheel fork with gear assembly and motor mount.

The fork of the wheel assembly in Figure 7 had to accommodate the motor shaft passing through and have a place to mount the motor itself. The motor mount has various reinforcement splines so that it is stiff and does not move under torque. All the nut pockets are designed for square nuts with spacers above so that they have more plastic area to grip onto.

**Figure 8:**
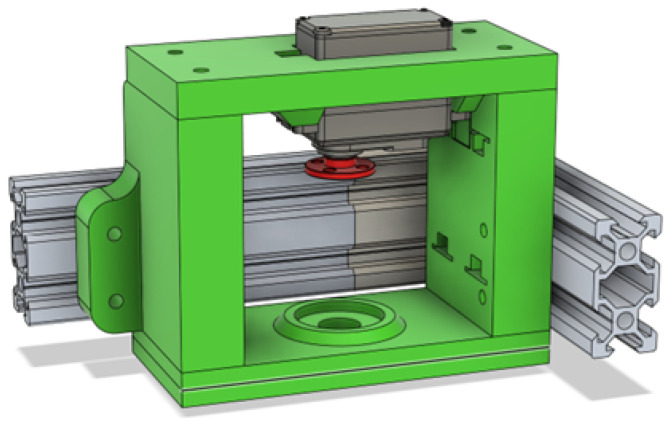
Wheel assembly frame mount.

The most complicated part of the wheel assembly was laying out the servo motor that is utilized for changing the direction and finding a good mounting for the fork on the frame. To that end the frame mount shown in Figure 8 was established. To hold the entire weight of the robotic platform and cargo, two sets of Axial Ball bearings were utilized in each wheel that squeeze together the frame mount using a long bolt.

**Figure 9:**
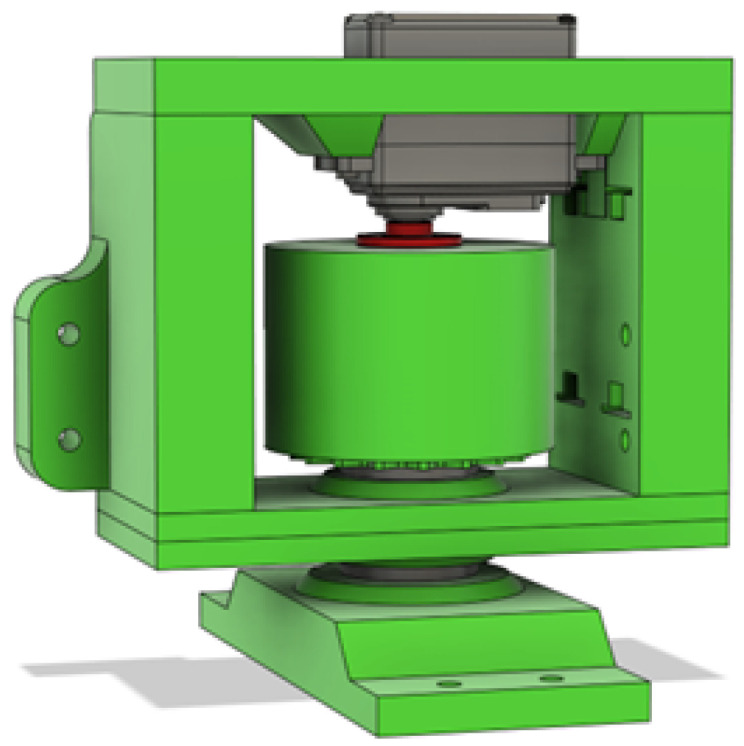
Servo motor coupling.

With this shaft completed, the servo motor needed to be attached to the new axle. For this reason, an interlocking star-shaped piece with a negative counterpart was designed so that the servo can be easily removed and to facilitate faster assembly and disassembly. The assembly can be seen in Figure 9. This turned out to be a great decision, since the servos needed to be changed to a different model along the assembly process to a faster and more powerful variant to reduce jitter and noise.

The inner star shape is fixed using two M12 nuts on the axle; the first one is a normal M12 nut, and the second one is a locknut. The outer star-shaped socket is fixed onto the servo using M3 screws, and the servo is fixed into its top plate using M4 screws that self-tap into plastic.

In this way, the full wheel assembly has both power and direction individually, separate from any of the other wheel assemblies.

**Figure 10:**
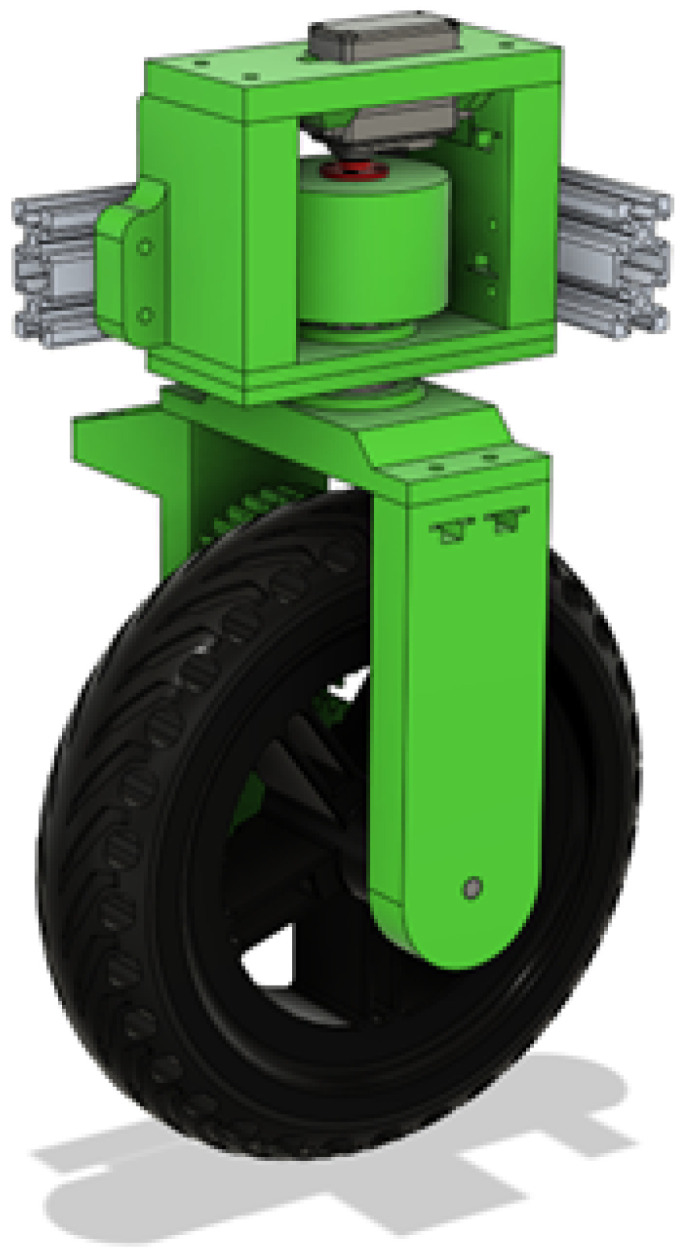
CAD render of the Independent Wheel Assembly.

The full wheel assembly in Figure 10 is a big system in itself that has to hold a quarter of the robot’s weight, so designing it was a thorough process that took into account every force that could act on it, from squishing plastic with screws to side-to-side loading to supporting a lot of weight while being able to rotate smoothly.

#### 3.1.3. Spool

The design of the spool is one of the most challenging parts of the entire project. One consideration was the need for it to be compact, reliable and fast. At first, a drum with only one layer of rope spooled on it sounded like the best idea, where each degree of rotation equaled a specific distance extended, but this soon turned out to be one of the most complicated versions, since the spool was being moved with a ball-screw on its axis to always align the rope with a single point of exit in order to guide it to the direction sensor. The other choice of just a normal spool was also not an option, since it is not feasible to perfectly lay rope on a spool, which means calculating the extended length of the rope would have been impossible.

The solution that proved to be the best one in this case was a spool that is of the same width as the rope. The rope is only one layer wide and creates a spiral when spooling. Calculating the length of the spiral is certainly possible. Additionally, there is a formula for approximating it with an error of 0.1%.

**Figure 11:**
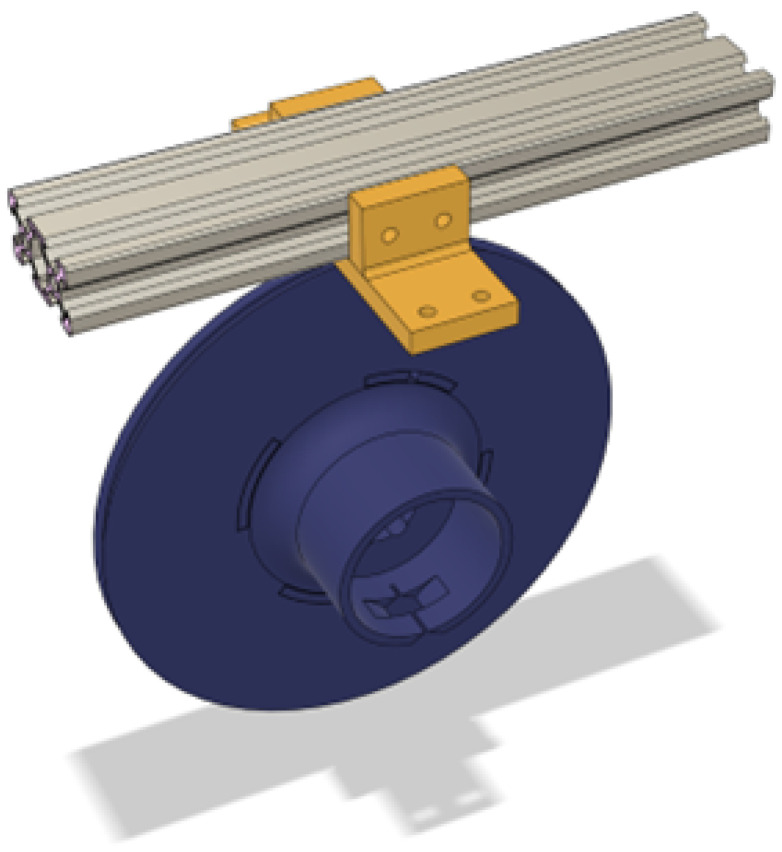
Spool top mounting bracket.

To also retract the spool, a flat spring that originated from a measuring tape was utilized. It is seated in the cylinder on the front side of the spool and is attached using a pin to the mounting arms to be able to apply tension to the rope. To mount the spool to the aluminum extrusion, a mounting bracket that supports the arms was utilized like in Figure 11. This bracket also has screw holes for both the sides and the bottom of the aluminum profile.

**Figure 12:**
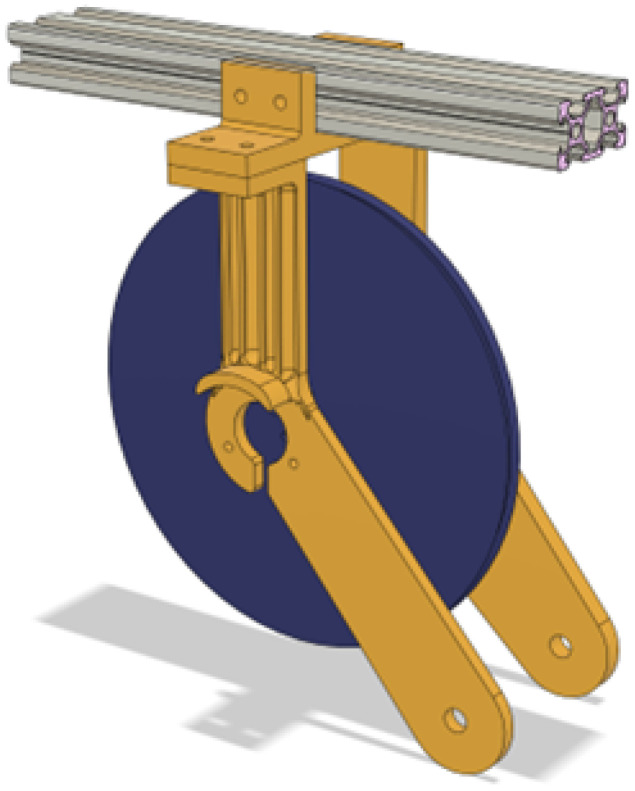
Spool mounting arms.

The spool then is held up by arms that extend further down to the alignment roller and on both sides have custom mountings for both the optical encoder and the pinion of the flat spring. The spool mounting arms can be seen in Figure 12.

**Figure 13:**
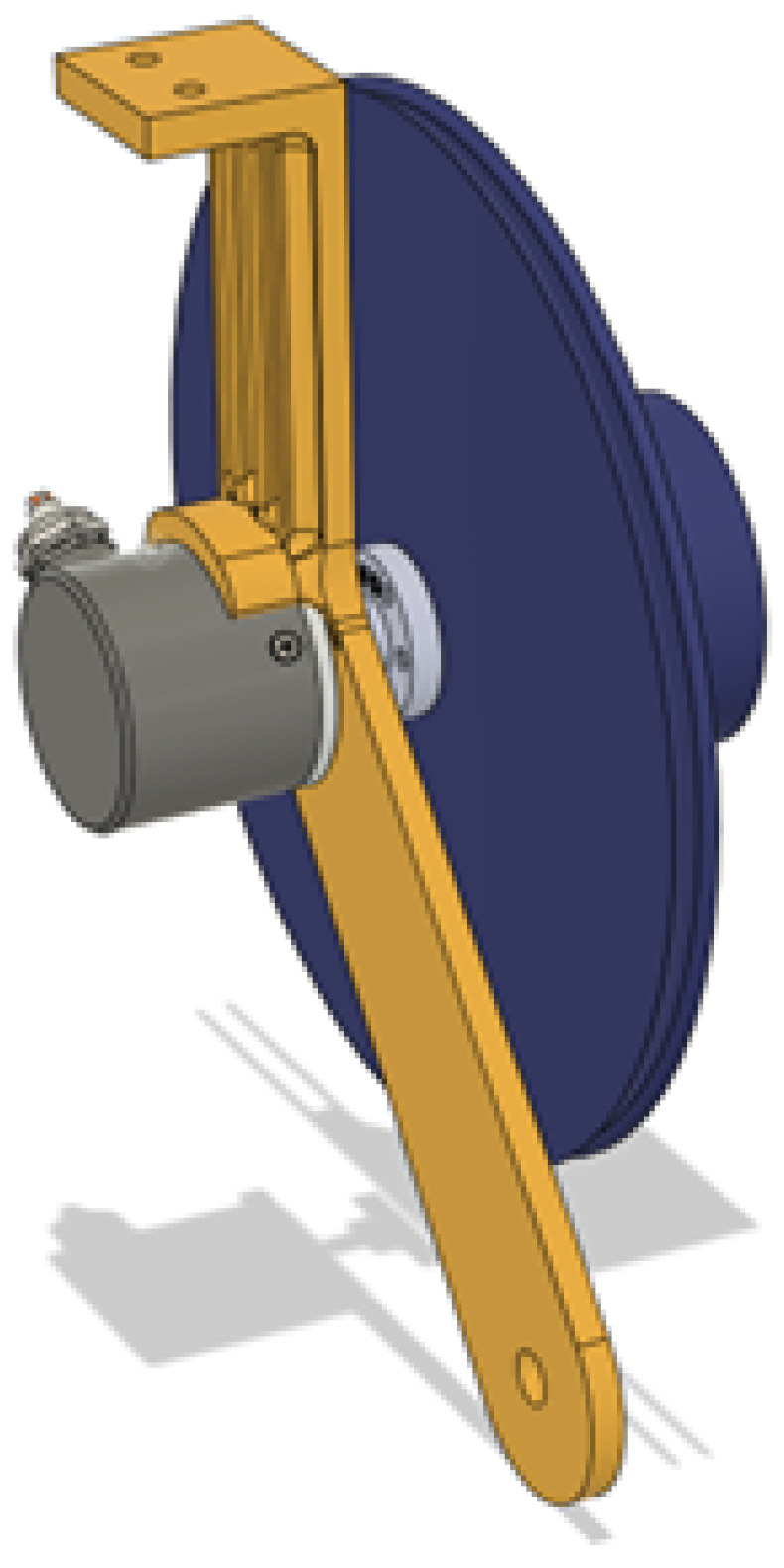
Optical encoder mounting.

The spool itself comprises two halves that mesh and screw together into the flange of the optical encoder (Figure 13). It has a slot for the rope to tie into a knot in the inner part of the spool before assembling it together so that it does not slip or come out even if fully pulled out.

**Figure 14:**
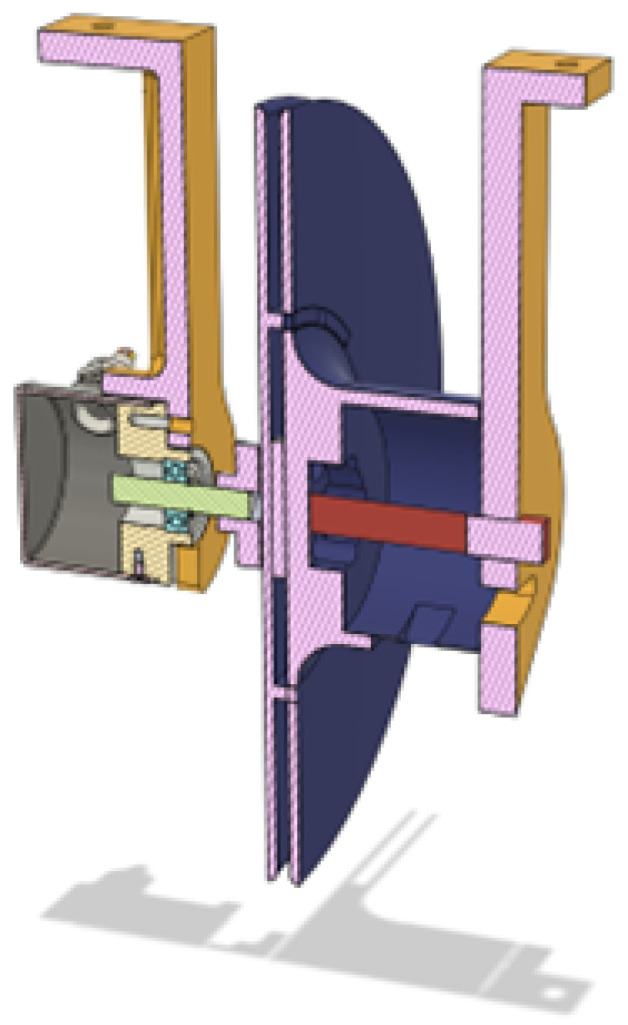
Cross-section of the spool assembly.

The flat spring is placed in the cylinder on the opposite side of the spool from the optical encoder, and the peg attaches to one end of it and ends up in a bearing for better stability. The peg has a bit of it protruding out since taking it out is a vital step in disassembly. The entire cross-section of the center of rotation of the spool can be seen in Figure 14, where the optical encoder, arms, spool and peg are presented.

**Figure 15:**
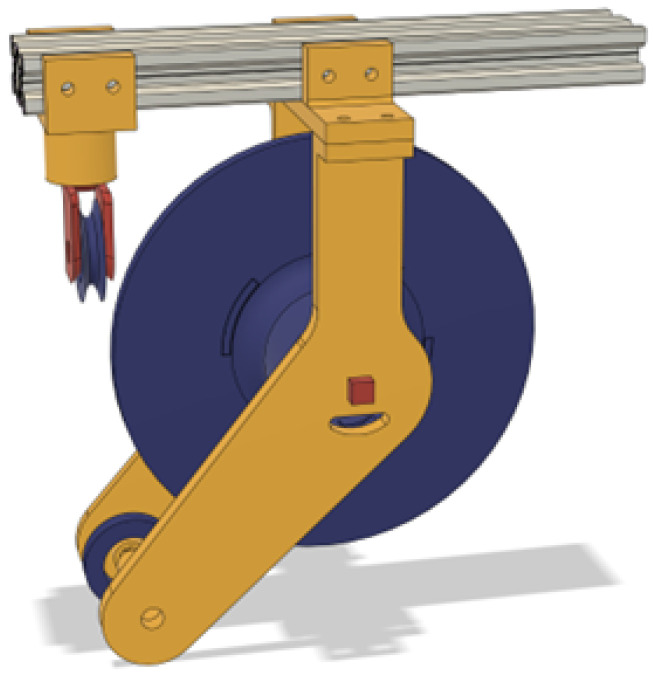
Directional roller with top mounting.

The alignment roller in Figure 15 is utilized to catch the rope as it exits the spool and puts it on a straight axis that then is utilized as a center of rotation of the direction sensor.

To sense the direction of the rope, a potentiometer was utilized. The potentiometer has its head stuck inside a bearing for smooth operation. The last piece would be the final roller with its fork, which is attached to the direction sensor within the bearing and fixed using an M2 screw.

The most complicated step of this direction assembly was the requirement for both the potentiometer and the fork for the direction roller to fit inside a bearing to ensure smooth operation and minimize the forces acting directly on the potentiometer when pulling the rope. The potentiometer being the center of the rotation gave a few challenges, especially with the thickness and strength of the materials. Another challenge with the potentiometer and material strength was the need to be able to insert the potentiometer in the part. That way a chamfered slot was designed that was barely big enough to insert the potentiometer while sliding and rotating it. The hole for its shaft was also chamfered for additional room.

The directional roller is operating smoothly, especially due to the alignment of the alignment roller, the alignment of the tangent coming from the alignment roller to the directional roller, and the rotational axis of the fork attached to the potentiometer. That way, in any way it rotates, the rope stays perfectly upright and does not change positions on the rollers, in any direction the rope extends.

**Figure 16:**
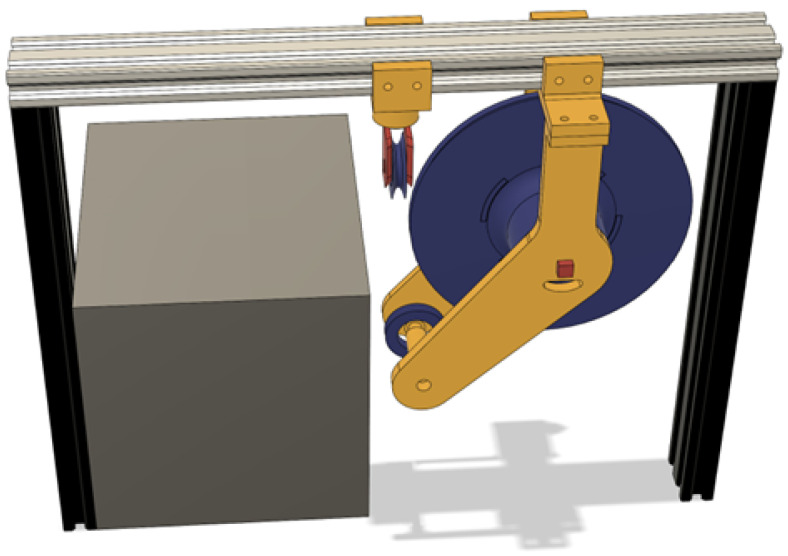
CAD render of the frontal area with the spool assembly installed.

While the roller needed to fit next to the battery and align perfectly, the space was a big constraint, as observed in Figure 16, and this was the lowest place it could be placed so that it could rotate toward the side with the spool.

### 3.2. Hardware and Electronics

Electronics are also a core system of the robotic platform. In order to control and sense all the parts of the robot, a lot of different components with various functions needed to be utilized, such as two types of motors, two types of encoders, motor drivers, voltage regulators, two types of microcontrollers and other parts, which I will describe more in-depth in the following subchapters.

### 3.3. Power Management

**Figure 17:**
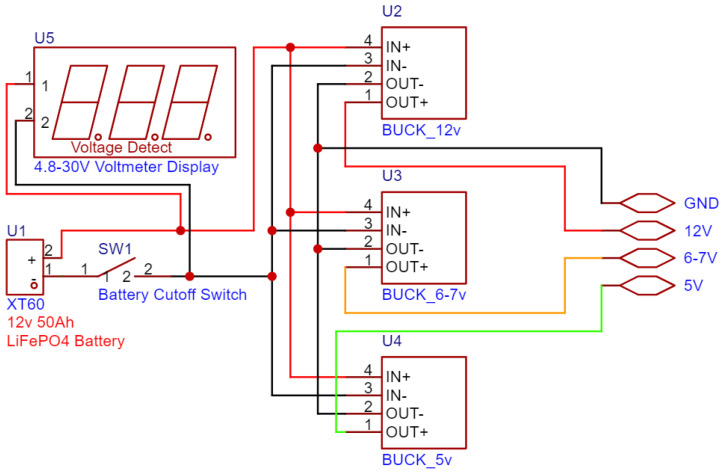
Circuit diagram of the power distribution system.

The robot is powered by a 12 v 50 Ah solar-grade LiFePO4 battery, like in Figure 17. The XT60 Connector was added to the battery so that it can be easily removed and utilized on other projects, but also for ease of use while charging. For charging, it uses an Andersen connector that came together with its counterpart on the charger. For safety reasons a 100 A battery disconnection switch was added, being then bundled together with a panel voltage meter to always see the voltage of the battery while powered on. These components are seated in another custom 3D-printed part.

The power that comes from the battery is then split between a 300 w inverter for powering the laptop that does image recognition and 3 voltage regulators that create 3 different voltage rails: 5 v, 6–7 v, and 12 v. The 5 v rail is utilized to power the microcontrollers and the 5 v-rated and tolerant components. The 6–7 v rail is utilized only for the servo power; that way it can be adjusted to give more or less power to the servo motors. Finally, the 12 v rail is utilized for the DC motors. These voltage rails are clustered together in the control section of the robot, next to the motor drivers and below the ESP32 Motor Controller Board.

#### 3.3.1. ESP32 Leader

**Figure 18:**
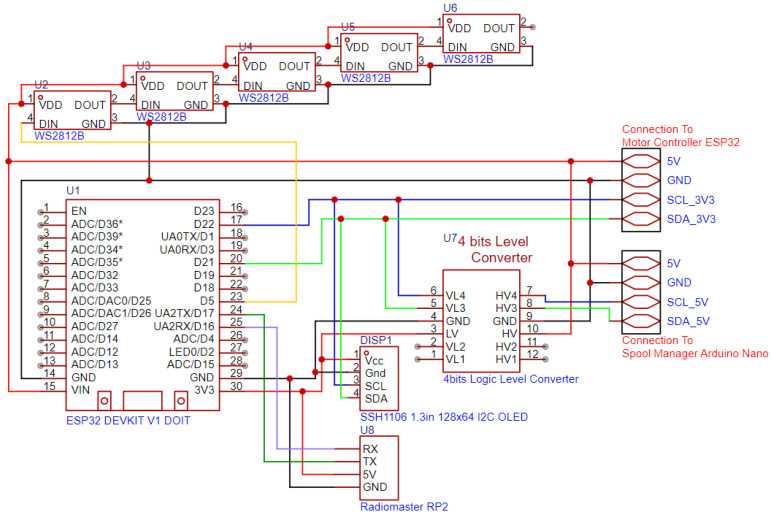
Circuit diagram of the ESP32 Leader and its external connections.

The ESP32 Leader (manufactured by Radiomaster, Shenzhen, China) is the ESP32 Board, as presented in Figure 18, which controls the entire robot and is connected to an ExpressLRS receiver, the RadioMaster RP2, via UART for communication with the remote control, and via I2C to the Spool Manager and the Motor Controller ESP32.

Since the Arduino runs on 5 v logic, the I2C bus is raised to 5 v on that section and communicates with the ESP32 Leader via a Logic Level Converter. On the other side of the I2C bus, the Motor Controller ESP32 is directly connected to the bus. The ExpressLRS receiver has 12 channels of data with values between 1000 and 2000, which it receives from the remote control and are utilized for state management of the robot; for example, arming and disarming the motors, switching between 2- and 4-wheel steering, and automation modes (spool-based and camera-based). All the states are remotely transmitted by the remote control; otherwise the data are invalid, and the robot does not move. This is a safety feature, to be able to always disarm the robotic platform from a distance. To visually see the various states of the robot, both the ones set remotely and the states of the connections with the other components over I2C and the link to the remote control, the ESP32 Leader has a monochrome OLED screen connected over the I2C bus and a set of 5 addressable RGB LEDs. The screen uses custom icons and progress bars to indicate states and values.

#### 3.3.2. ESP32 Motor Controller Board

**Figure 19:**
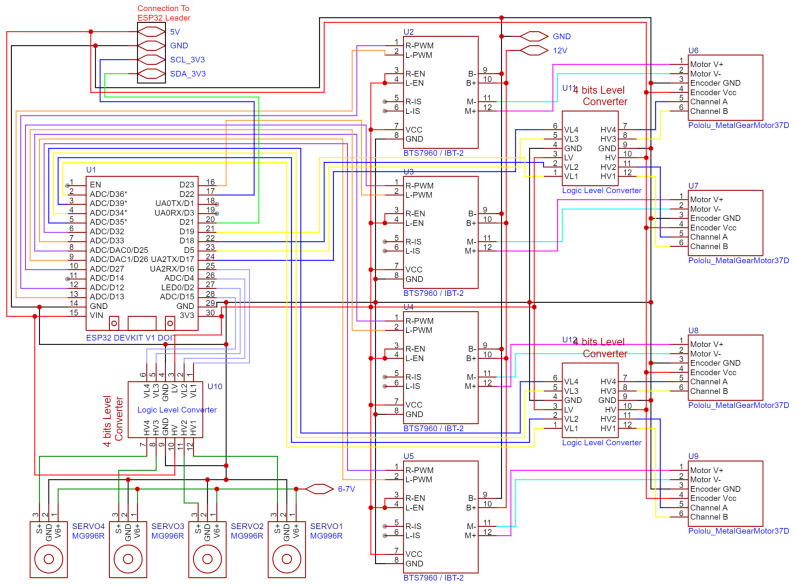
Circuit diagram of ESP32 Motor Controller, motors, drivers, external connections.

The Motor Controller Board has most of its resources used up, as is clearly seen in Figure 19. Almost all the GPIO pins, timers and processing power are utilized for controlling and reading the motors and calculating independent angles, speeds and feedback from each wheel. To that end, it is connected to 4 servo motors, 4 motor drivers that are connected to the DC motors and 4 encoders attached to those DC motors. Additionally, it runs four independent PID loops, which will be described more in the Embedded Code chapter.

The ESP32 uses a 3.3 v logic level, so for interfacing with the servos and the DC Motor Optical Encoders, 3 four-channel Logic Level Converters were utilized. The servos need one channel each for the 5 v PWM control signal, and the optical encoders need two channels each for both phases, which also operate at 5 v minimum. Almost every cable that connects this board to external components is connected with manually terminated 2.54 JST XH connectors to ensure the orientation of the connector except for the motor driver boards which use DuPont Headers. The board receives power from the 5 v rail powered by the voltage regulator and sends it along through the I2C bus connector to all the other boards. Additionally, it receives the 6–7 v rail and splits it to all the individual servo motors on the wire harness. In order to recognize and organize all the connections to the motor assemblies, they were labeled and bundled together into a harness. These cables route to all the corners of the robot and are supported by 3D-printed clips that attach to the aluminum extrusion slots. There were many considerations for using the normal ESP32. The ESP32-S3 was utilized for this board, but it turned out to have too few LEDC timers, utilized for motor control, both on DC motors and servos.

#### 3.3.3. Spool Manager Arduino Nano

**Figure 20:**
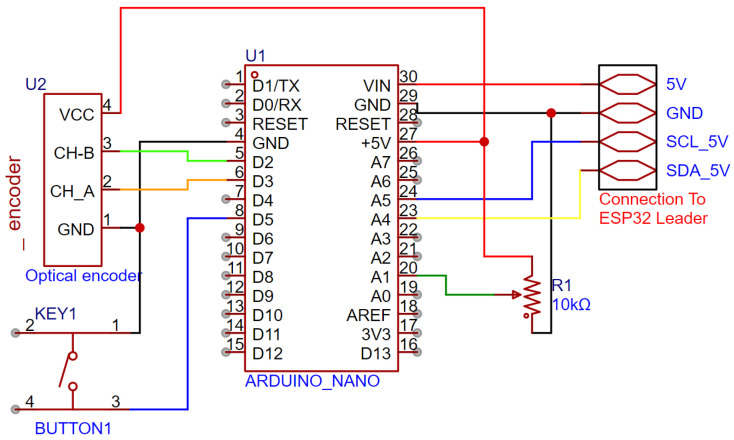
Circuit diagram of the Spool Manager Arduino Nano and external connections.

To reliably read the Spool Extension Sensor and the Direction Potentiometer, a separate Arduino Nano was utilized. This board had to have resources available for interrupts and run at the 5 v logic level in order to read the optical encoder that is rated between 5 and 24 v. Unfortunately, that optical encoder uses a 7805 series voltage regulator, so it is in fact not rated at 5 v. The solution to this issue is solved by disassembling the optical encoder and bypassing the 7805-series voltage regulator. With this modification to the encoder, the Arduino could reliably read the encoder. The circuit diagram for that can be seen in Figure 20.

The potentiometer is being read by one of the analogic inputs of the Arduino and interpreted as degrees. To communicate with the ESP32 Leader, it uses the 5 v section of the I2C bus. The Arduino requires a calibration sequence to calculate the extension of the rope correctly. The calibration is triggered by a button connected to a digital GPIO on an internal pull-up resistor. The calibration sequence is started by pulling all the rope out from the spool, pressing the button once, releasing the rope back into the spool and pressing the button again. This way it records how many degrees the optical encoder rotates to store the entire rope on the spool.

### 3.4. Embedded Code

The architecture of the system is based on one leader and two followers on the I2C bus. The leader receives requests and sends all the communication so that the robotic platform runs smoothly; it communicates with the ESP32 Motor Controller Board and the Arduino Nano Spool Manager over the I2C bus and with the laptop running the image recognition code and sending the processed data over USB.

**Figure 21:**
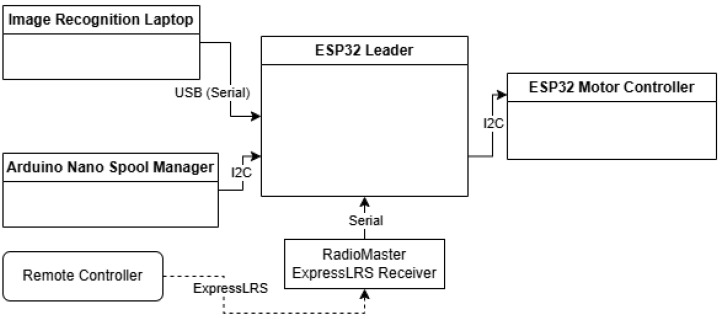
Diagram representing the types of connections between the systems.

The diagram in Figure 21 shows the various communication protocols and connections utilized to power the robot and to transfer the data between all the systems.

#### 3.4.1. Program for ESP32 Leader

**Figure 22:**
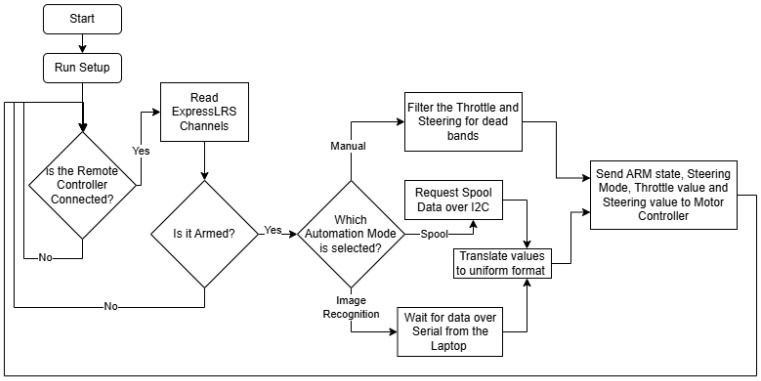
Flowchart of the ESP32 Leader code.

The ESP32 Leader makes all the decisions and handles all the states as dictated by the remote control; a representation of its program can be seen in Figure 22. It receives messages from the remote control over the ExpressLRS protocol using a dedicated receiver through UART. This way it has three modes of operation: manual mode, spool-driven and image recognition-based. Additionally, it has two ways of steering available: 2-wheel steering and 4-wheel steering.

In the diagram above you can see the general overview of how the ESP32 Leader operates, how it handles the selected modes, states and data for direction and steering, and how important the ExpressLRS connection is, to the point where it will not run if the remote is not connected. Speaking of the ExpressLRS receiver connection. The ESP32 is one of the lowest-end microcontrollers that can handle this kind of data throughput. It needs to run the UART communication with the receiver at a 400,000 baud rate to be effective since the ExpressLRS protocol sends packets at a rate of up to 500 Hz while in use on the RadioMaster TX16s MKII Remote Controller. ExpressLRS runs on the CrossFire protocol, which is why the library needed to decode and interpret these packets is the crossfire serial library. On the I2C bus there is also an OLED screen that is utilized to display all the states and modes through custom icons that denote the current values, to see at a glance, directly on the robot, how it will react to inputs. The way it operates is by using a constant check for connection to the remote control; if not connected, it will not send commands or requests for data to the followers. Then it has three time-based loops that only run at certain intervals so that it does not overwhelm either the display, the addressable LEDs (referred to as “pixels” in code, from their name: NeoPixels), or the I2C bus with commands to the Motor Controller Board. The display loop runs every 30 ms. Show all the icons and gauges for the states by displaying custom bitmaps based on the values received from the ExpressLRS receiver or internal states. The data that is displayed is as follows: steering input and throttle input as gauges; ARM state, I2C connection state, automation mode, and steering mode as icons.

The Pixels loop runs every 50 ms to show the color of the current state: red for disconnected, turquoise for connected but disarmed and green for armed. The Motor Controller Board loop runs every 20 ms. It sends filtered information to the Motor Controller Board. A big reason the inputs need filters is the accuracy of the input sticks; dead-bands were implemented in the most common areas so that it does not jitter when the sticks are in edge cases such as the center and ends of the travel. With that in mind, the ESP32 Leader sends the following data to the Motor Controller Board: arm state, steering mode, throttle and steering. With each different automation mode, it sends those pieces of data based on different sources. One of the sources is the Spool Manager Arduino Nano, which acts as an I2C follower. The ESP32 Leader needs to request data from it in the form of how many millimeters the rope is extended and at how many degrees the rope is in comparison to the robot, with the center at 0 degrees. These are requested over I2C right before sending the data to the Motor Controller Board.

For filtering and routing the spool data to the motor controller, the ESP32 Leader first requests that data, then translates it for following the person and sends it through I2C to the ESP32 Motor Controller. This is performed in the same way with the serial input received from the laptop, but with different translation values and limits.

The other source is the image recognition over serial, where it receives two comma-separated values: direction and distance in millimeters. In case of a failure to determine the distance or direction, the image recognition code will send 0.0 so as not to move the robot when it cannot determine exactly where the person is.

#### 3.4.2. Program of ESP32 Motor Controller Board

**Figure 23:**
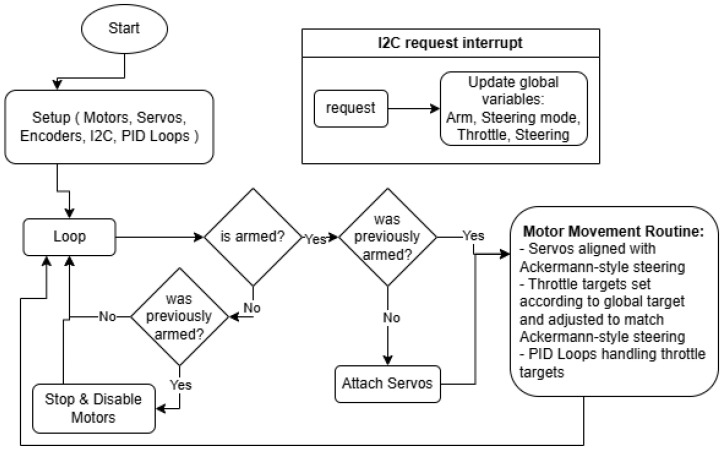
Flowchart of ESP32 Motor Controller code.

The ESP32 Motor Controller is an integral part of the robotic platform due to its direct control over the movement. A lot of precautions had to be taken so that it is well implemented since many of the mechanical parts are out of 3D-printed plastic; although strong, they are not as strong as the aluminum structure. One of the big considerations was that the wheels are not allowed to slip; because they are rubber, they would grip the road surface hard and could pull the robot apart. Thus, Ackerman-style steering was implemented, with each wheel having an individual angle and individual speed. In short, Ackerman steering refers to the fact that when the robot steers, due to its width, it has its wheels on two concentric circles, so both the speed and the angle of the wheels differ from each other. This creates a sort of virtual differential drivetrain. The program representation can be seen in Figure 23.

For ease of use, all the motor assemblies have initializations of pins and settings for the DC motors, servos, and other constants in the same place.

There was also the need to test how it handles 2-wheel and 4-wheel steering, so to differentiate, parameters for the steering algorithm were implemented. And to compute the Ackermann model, it needed the concentric circles and tangents, which represent the wheels and speeds for each wheel. Below is a left turn example.

With this implementation the wheels do not slip anymore, and the advantages of all-wheel power can be seen. The other big consideration was that the ground beneath the robot could be uneven, or it might encounter slight obstacles. For this reason, a PID loop was implemented for each individual wheel; that way, when encountering an obstacle with even only one wheel, it will correct and try to compensate with the throttle for the target speed of the wheel. The PID Library utilized is created by Dlloydev on GitHub 3.16.2 and has many useful features [22]. The loop, in fact, is only PI, since there is a little bit of play in the gears, so the D-term, instead of dampening, both under-corrects and over-corrects at times.

The way the PID loop obtains its input is through the internal encoders of the DC motors. The physical measure and target for the PID loop is speed; that way it does not account for the distance traversed but instead its derivative.

#### 3.4.3. Program of Spool Manager Arduino Nano

**Figure 24:**
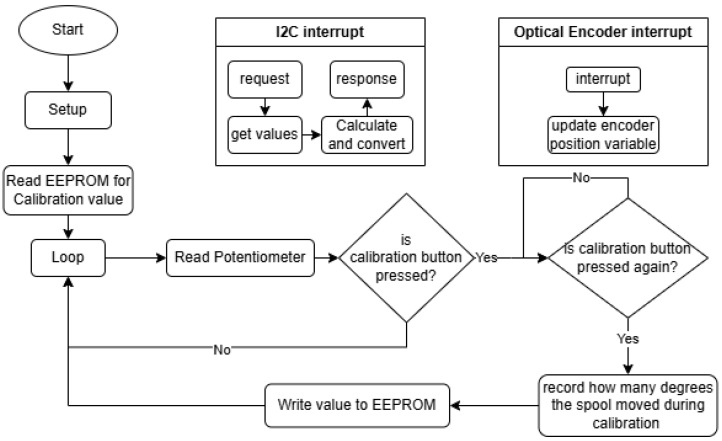
Flowchart of the Spool Manager Arduino Nano code.

The Spool Manager Arduino Nano handles a few tasks at a time, as shown in Figure 24, such as interrupts for the spool optical encoder, reading the potentiometer that the direction of the rope obtains, and handling I2C request events from the ESP32 Leader.

Reading the spool is achieved by triggering the interrupt pins, which increment or decrement the value of the variable that handles the state of the spool.

This would be without significance if the spool was not calibrated, so a button can be utilized to trigger the calibration sequence, which has to be performed in the following order: Pull the whole rope out of the spool; press the calibration button; release the rope back into the spool while holding onto it; and press the calibration button again to indicate finishing the calibration sequence.

The calibration sequence accomplishes a single task: it records how many degrees the spool turns for hosting the entire rope; this way it can calculate the length of the extended rope just based on the internal diameter of the spool, the diameter of the rope, accounting for the squishing while spooling and the number of degrees it turns while unspooling. The spiral length equation for the spool-based tracking system was deduced from two other equations found on a peer-reviewed online calculator [23]. Equation (1) indicates the length of the rope L, based on the inner diameter d, outer diameter D, and number of turns N.

(1) $$L=\frac{\pi \times N\times (D+d)}{2}.$$

Equation (2) indicates the number of turns N based on the inner and outer diameters and the thickness of the rope.

(2) $$N=\frac{D-d}{2\times t}.$$

This helped deduce Equation (3), which takes out the outer diameter and only uses the number of turns N, the thickness of the rope t, and the inner diameter of the spool d.

(3) $$L=\pi \times N\times \left(t\times N+d\right).$$

This equation, which approximates the spiral formula, is accurate with an error of about 0.1% when compared to the original, as stated in [23]. Below is the implementation of this formula in code.

To retain this information after powering down, this value is written to EEPROM and is read at setup time.

To calculate the amount of rope out of the spool, the difference between a full spool and the amount left on the spool was computed.

When the ESP32 Leader requests the data, the information is assembled into a packet and sent, since the Arduino Nano always has the most up-to-date spool and direction data stored as global variables, available at any time.

The packet is formed this way to make I2C transmission easy. On the receiving side it needed to be parsed with a mask, since the two controllers have different architectures and represent variables in different sizes in memory.

### 3.5. Image Recognition Code

The image recognition is performed in Python 3.13.3 with the help of the YOLOv11 pose estimation model [24]. A simpler object detection algorithm would not have sufficed for calculating distance, since a person is rarely seen from head to toe in an image if the camera gets close; thus, it needed a different approach, which pose estimation offers. With pose estimation, the YOLOv11 model gives the positions of joints and other key features in a human body; among those are the eyes, nose, ears, shoulders, elbows, hips, knees, etc. The data relevant to this case is the position of the hip joints, since the camera is at hip height and other areas might not be visible at close-up distances.

Based on the average x-axis positions of the hip joints in the image, the direction can be determined. These translate to values from −100 to 100, with 0 being the center of the screen. To estimate the distance from the camera to the person, the distance between the hip joints can be utilized. To translate this measurement to a physical dimension, an initial calibration is needed. When standing 2 m away from the robot, the distance between the hip joints is measured in pixels and introduced in the code as a constant. Using the calibration, the distance output is expressed in millimeters.

When the confidence score of both hip joints is high enough, and they are both present in the image, the program sends both the direction and distance over USB to the ESP32 Leader as a comma-separated pair of integers. On a battery-powered laptop running an NVIDIA RTX 3060 laptop graphics card, the processing takes between 25 and 35 ms. This gives the feeling of smooth tracking when the subject is not lost from the frame, or a misinterpretation of the image takes place.

## 4. Results

**Figure 25:**
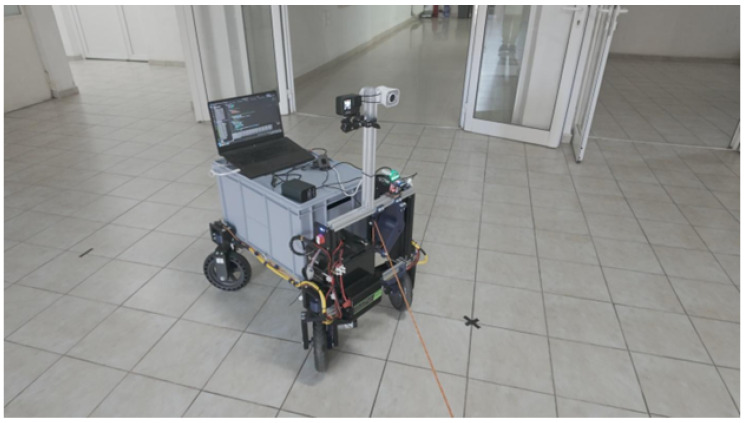
Image of the robotic platform with all tracking systems running while mid-turn.

There are a few mentions about methodology that need to be stated before proceeding with the results. For performing the experiments, the floor was marked every 2 m along a square perimeter that was 4 m long by 4 m wide, with the center marked as well. Before the experiments, calibrations were run for both systems. Before running the experiments themselves, a few rounds of arbitrary following were accomplished, like in Figure 25, to see how the robotic platform and the systems perform on the site of the experiment. Before each experiment, the logging was started for all systems with relevant data and stopped at the end of the experiment. All wheel assemblies have servos to which data in degrees is sent and DC motors with encoders that can record their physical rotation. The spool also has a dedicated Arduino Nano, which was connected via serial to the laptop performing the data logging and image processing, which also recorded that data. This constitutes three sources of data: The first one represents the robot’s physical location in the world over time, which was utilized to represent the path taken; the second one represents the spool tracking data, which represents the rope extension and the extension direction; and the third source is the image processing data, which also represents the distance to the person it is tracking and the direction.

The spool is truly accurate; it is accurate to ±5 mm, an error that can be attributed to cable squishing, and does not skip steps. The spiral formula is calculated well, and it can be seen by comparing a measuring tape to the software results.

### 4.1. Path Comparison

**Figure 26:**
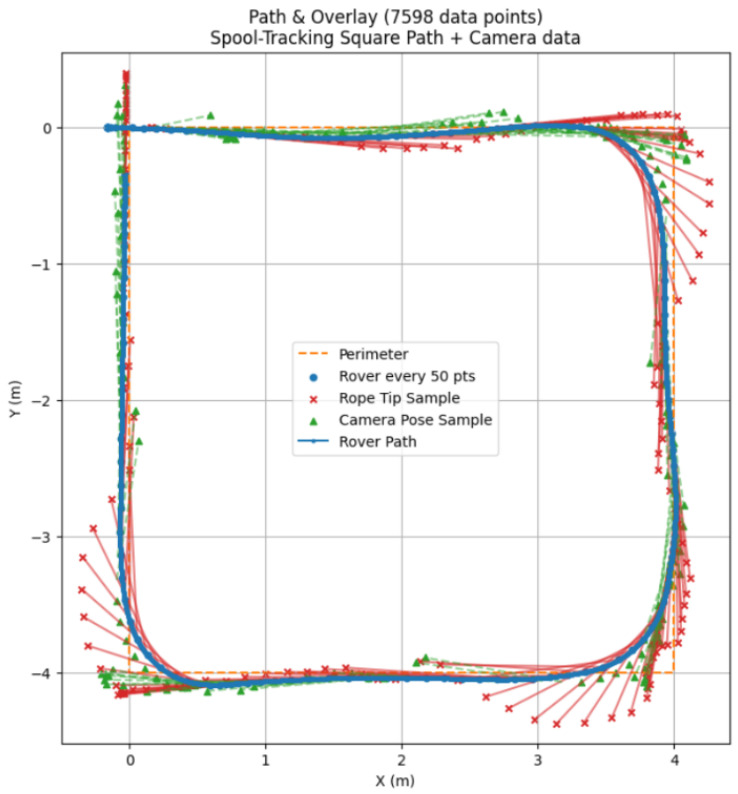
Plot of the physical path of the platform using spool-based tracking with overlays.

The spool-based system tracks accurately with tight following of the person as seen in Figure 26, and the subject is more comfortable when stopping, since the spool does not lose track of the subject when up close. The camera-based system can lose track of at least one hip joint when up close and, although it stops, confidence in this system is generally lower.

**Figure 27:**
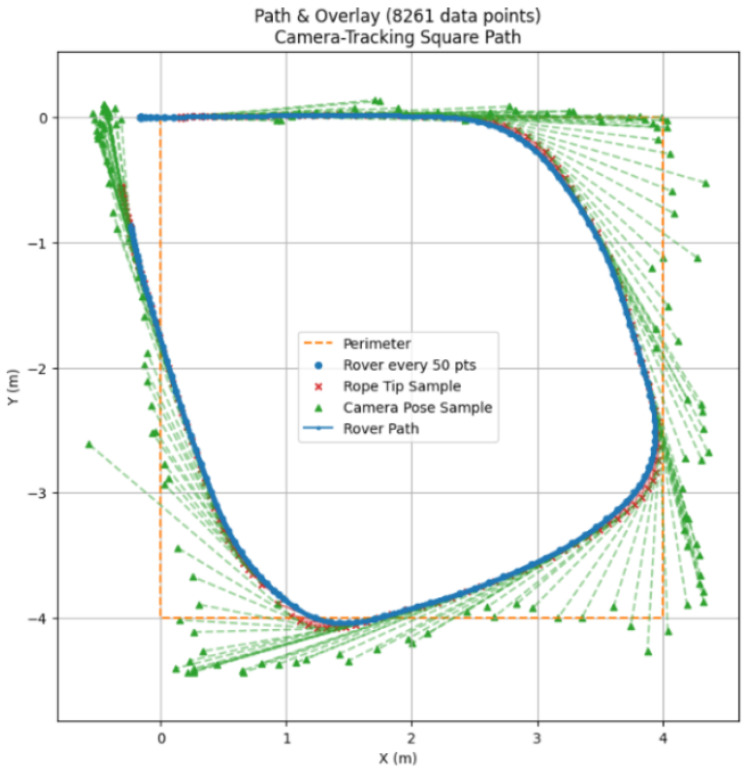
Plot of the physical path of the platform using camera-based tracking with overlay.

In Figure 27, it is evident that the path following of the camera-based tracking system is considerably less responsive to changes in direction due to its lower angle of view when compared to the spool-based system.

Although it seemed at first that image recognition would be way worse at estimating distance, it performed well. It is not as accurate as the spool, and the tracking data are noisier since it must estimate where the hips are, but since this is performed at an interval of about 30–40 ms, it is quick enough to appear continuous. The Path plot can show that the tracking did not lose track of the subject, but at the edges of the frame it overestimates distance significantly.

### 4.2. Accuracy, Error and Reliability Comparison

**Figure 28:**
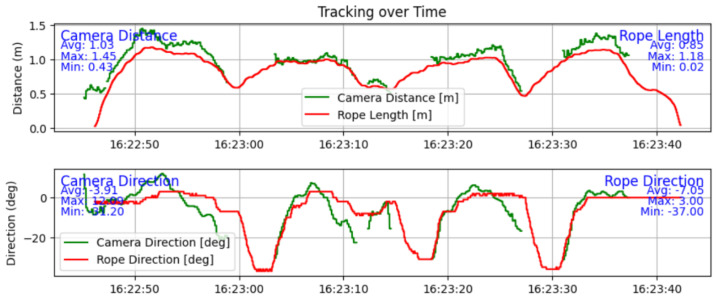
Graphs of the distance and direction values over time of both tracking systems.

A surprising finding is that the YOLOv11-based pose estimation is more responsive at determining direction than the spool-based tracking system since there is a mechanical limitation on the direction roller of the spool. The results presented in Figure 28 show that the camera’s lower angle of view makes the tracking system lose the subject from the frame, as evidenced by the missing sections of data. In Figure 28, it can also be seen that due to the mechanical nature of the spool-based system, it has a lag in determining the direction of movement, between 0 and 2 s depending on the magnitude of the turn. This mechanical disadvantage can be easily reduced in a later iteration.

**Figure 29:**
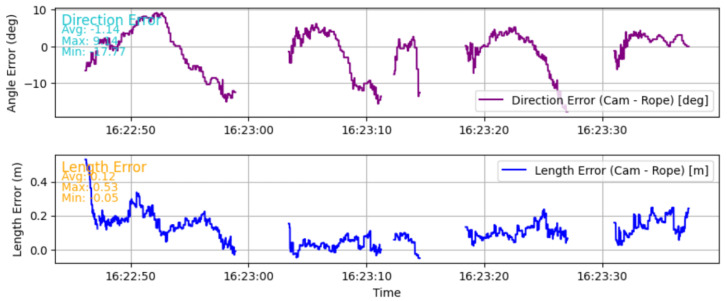
Graphs of the error in direction and distance between the systems over time.

The average error between the length estimations is 12 cm, as seen in the text of Figure 29, which shows that both systems, when calibrated and started correctly, are quite performant. The maximum error is 53 cm in this experiment, and it shows that the camera-based system is more prone to wrong estimations. The error in direction is different; looking again at Figure 29, with the biggest absolute difference of 17.77°. This error comes from the lag of the spool-based tracking system. The absolute average error is 1.14° instead.

**Figure 30:**
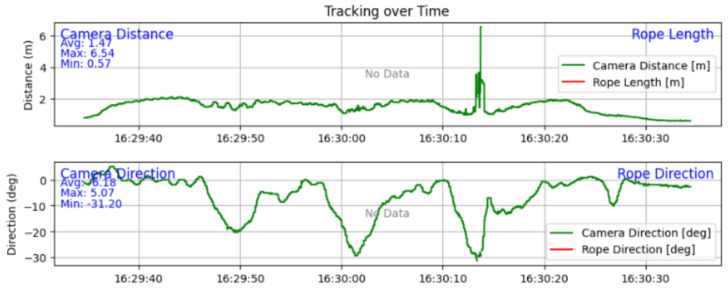
Graphs of the distance and direction of the camera-based system when tracking.

A finding in reliability can be seen in Figure 30, where the distance estimation has a spike when the subject is close to the edge of the image frame. This exemplifies the lower reliability of the camera-based tracking system.

The last finding of these experiments is that while the camera distance estimation is quite good, it is noticeably noisier than the spool-based length, though. One other downside of the camera-based system is that it also loses track of the subject easier and needs a longer time to adjust the robotic platforms’ direction so it tracks the subject; thus, it is less responsive overall.

### 4.3. Comparisons with the State-of-the-Art

Most of the referenced papers discussing human following did not suggest or state measurements regarding the tracking systems. The papers [3,7,8] mention error of measurements; the paper [12] measures responsiveness of back-and-forth motion; and finally, paper [13] shows both the path taken and the average distance of the robot from the user. Compared to paper [7], both spool-based and camera-based tracking systems appear to perform better in smoothness. Where the robot in [7] oscillates, the mobile robotic platform does not, especially on the square. Unfortunately, comparing error with this paper seems unproductive, since the author does not mention how they measured all the error components, specifically the human position.

For tethered tracking, paper [2] presents a plot that compares the two trajectories of two robots, which are similar. This can also be said about the mobile robotic platform using spool-based tracking, which appears to be smoother than the difference between the robots in paper [2]. Additionally, in the results presented before of the spool-based tracking, it can be seen that the robot keeps a following distance between 50 cm and 1 m while in the middle of tracking, with an average of 85 cm following distance. Which is also close to the average between participants of the minimum comfortable following distance of 80.12 cm, as determined by paper [11].

The difference in following distance, as described by paper [13], between the robot and human is 10 cm during their experiment, with a fixed 50-centimeter following distance. In the case of the mobile robotic platform while utilizing the spool-based tracking, the following distance is dynamic, with increased speed at further distances and reduced speed when close-by. This results in a following variation of about 50 cm, which is between 50 cm and 100 cm following distance, including trajectory changes and speed changes. While tracking using the camera-based system, it can be seen that the variation is about 1 m, while the tracking distance is between 1 and 2 m. This can be attributed to the need to be in the frame while performing right-angle turns on the square perimeter of the experiment. When compared to paper [13], it can be seen that the variation is larger, but that is due to two factors: the above-mentioned dynamic following, which is needed because of the size and inertia of the robotic platform, and the fact that the robotic platform is four-wheeled and needs to move in distance as well when following a person, which affects the camera-based following, where the frame is narrower.

While the provided quantitative evaluations highlight tracking’s accuracy and reliability, they also highlight the different nuances of human–robot interaction. The sensing methods align with different behavioral requirements. Spool-based tracking systems can be utilized in higher-distance maintenance scenarios, supporting the perceived feeling of safety, but they lack fluidity of movement, providing an overall less comfortable experience. Camera-based tracking adds the possibility of capturing subtle body movements or even facial expressions, making it more reliable for anticipatory movement adjustments, but it remains more limited regarding lighting variations or possible camera occlusions. The sensor fusion approach combines both of the previous methods’ strengths: being at the same time the most robust system and the most promising technology for the alignment of the robotic system to social acceptability and norms. This method allows the robot to follow the user at comfortable distances with robust continuity while also integrating specific semantic cues.

From the perspective of proxemics, the ability of the system to regulate the distance while following the user is critical. Our proposed comparisons show that Spool excels at keeping equal distances during movement but lacks the ability to contextually adjust to the needs of the user, lacking the cameras’ semantic flexibility. On the other hand, camera-based systems may not be stable under poor visibility conditions, the robot potentially lacking in responsiveness. Sensor fusion offers the possibility to mitigate the previously described faults of the system by supporting both functional reliability and better adaptive behavior. These observations suggest that the fusion-based approach is best aligned not only with reliability in real-world following situations but also with human comfort.

## 5. Conclusions and Future Work

The two systems have both advantages and disadvantages over each other; while camera-based tracking has a faster responsiveness when it comes to direction, the spool-based system has the advantage of reliability when it comes to determining the distance to a person. While the disadvantage of the camera system is that it is generally less responsive when combining all the factors and tends to make bad estimations, the disadvantage of the spool-based system is the need for a physical connection to the person it is following. With all this being said, both systems have a place. When in crowded areas, the physical connection of the spool-based system is a good indicator that the robotic platform is following its user, and a person will not get between them. Additionally, the camera-based system might struggle there to identify the correct person. On the other hand, when using the platform in a sparsely crowded area or with no one around, the user might prefer a system that can follow without a connection so they can rotate around freely.

One of the first ways to improve the tracking, in general, would be through sensor fusion; if both systems give the same parameters at the same time, this could be a good way to use all of the sources for these physical measurements and combine them so that it becomes more reliable and responsive. Sensors’ implementation may also be enhanced by the previously exposed tracking systems in order to complement potential malfunctions due to noises, deformations or disturbances. The robotic platform is extremely expandable and made to be able to carry cargo. This means that it can be modified with additions such as a robotic arm, solar panels, a better drivetrain and others.

There are two directions this platform could be improved: one software and the other hardware. The software improvement could include path following instead of directly following a person, so, for example, when taking a corner, the robot will not directly go into the corner but follow the path that the person took. This can be accomplished by using an array of points in 2d space, which the robot follows and adds upon when the user moves. The other way of improvement would be in addition to holding cargo, it could handle said cargo, so it can further add to its autonomy. An example that comes to mind would be the ability to pick up items from the ground. Another way to further expand upon this platform would be to add environmental sensors and make it a data collection powerhouse equipped with GPS that would record parameters about the world around it.

In the following Table 1 the referenced tracking systems can be seen compared to the implemented solution; while few systems are on large platforms, some systems are able to have a lower theoretical error, and none compare two independent systems on the same physical platform. Especially the comparison of a fully internal system like pose estimation and an external system like tethered tracking is important since it can show how specific parameters of each system behave compared to another.

In addition to providing accuracy and reliability, future developments of human-following robotic systems must also conform to social and behavioral paradigms. Tracking systems should not focus only on following users but also provide a comfortable, socially acceptable and predictable user experience. Our descriptive comparison highlights the Spool systems’ ability to precisely control their following distance and the richer behavioral cues the camera-based systems provide while also showcasing the advantage of integrating sensors into human-tracking robots.

## Figures and Tables

**Table 1:** Comparison table indicating the differences between the proposed system and existing systems in the literature.

Reference	Tracking Type	Platform Size	Tracking Error	Tracking Systems
[1]	External	Medium	-	Tether
[2]	External	Small	Low Error	Tether
[3]	Internal	Medium	99.5 cm	IR Beacon
[4]	External	Small	-	IR Beacon + Line
[5]	External	Small	High	Wi-Fi RSSI
[6]	Internal	Large	-	Image Recognition
[7]	Internal	Small	6.8–15.3 cm	Image Recognition
[8]	Internal	Medium	-	Pose Estimation
[11]	Internal	Medium	-	LRF
[12]	External	Small/Large	100% task success rate	Image Processing
[13]	Internal	Large	5.6 cm	LRF
Our Solution	Internal/External	Large	12 cm/1.14 deg	Tether/Pose Estimation

## Data Availability

The original contributions presented in this study are included in the article. Further inquiries can be directed to the corresponding author.

## References

[1] Fukushima E.F., Kitamura N., Hirose S. (2001). Development of tethered autonomous mobile robot systems for field works. Adv. Robot..

[2] Na S., Ahn H.-S., Lee Y.-C., Yu W. (2009). A tethering device for mobile robot guidance. Int. J. Adv. Robot. Syst..

[3] Arbula D., Ljubic S. (2020). Indoor localization based on infrared angle of arrival sensor network. Sensors.

[4] Ma R. Line following and beacon tracking robot based on Arduino Mega 2560. Proceedings of the 2021 3rd International Symposium on Robotics & Intelligent Manufacturing Technology (ISRIMT).

[5] Yu Y.-S., Ke C.-H., Chen Y.-S., Yu P.-Y. Development of following vehicle prototype using robot operating system. Proceedings of the 2019 8th International Conference on Innovation, Communication and Engineering (ICICE).

[6] Yong C.L., Kwan B.H., Ng D.W.-K., Sim H.S. Human tracking and following using machine vision on a mobile service robot. Proceedings of the 2022 IEEE 10th Conference on Systems, Process & Control (ICSPC).

[7] Jommuangbut J., Sritrakulchai K. Development of the human following robot control system using HD webcam. Proceedings of the 2018 International Electrical Engineering Congress (iEECON).

[8] Ha E.-J., Choi J.-Y., Son M.-J., Jeon J.-H., Kim A.-H., Baek S.-J. Development of a gait measurement system based on a human-following mobile robot. Proceedings of the 2023 23rd International Conference on Control, Automation and Systems (ICCAS).

[9] Li W., Passama R., Bonnet V., Cherubini A. A comparison of human skeleton extractors for real-time human-robot interaction. Proceedings of the 2023 IEEE International Conference on Advanced Robotics and Its Social Impacts (ARSO).

[10] Reddy V.S.R., Chintala K.S., M. A., S. V. A vision-based gait analysis and fall detection using YOLOv11 model. Proceedings of the 2025 International Conference on Wireless Communications Signal Processing and Networking (WiSPNET).

[11] Chebotareva E., Magid E., Carballo A., Hsia K.-H. Basic user interaction features for human-following cargo robot TIAGo base. Proceedings of the 2020 13th International Conference on Developments in eSystems Engineering (DeSE).

[12] Kanchanasatian K. A robot companion algorithm for side-by-side object tracking and following. Proceedings of the 2022 37th International Technical Conference on Circuits/Systems, Computers and Communications (ITC-CSCC).

[13] Petrov P., Georgieva V., Kralov I., Nikolov S. An adaptive control scheme for human following behavior of mobile robots. Proceedings of the 2020 XI National Conference with International Participation (ELECTRONICA).

[14] Xu T., Ma S., Xu H., Mo S., Li Y. Application of Ackermann steering in obstacle crossing platform of six-wheeled robots. Proceedings of the 2023 2nd International Symposium on Control Engineering and Robotics (ISCER).

[15] Ge P., Guo L., Chen J. Electronic differential control for distributed electric vehicles based on optimum Ackermann steering model. Proceedings of the 2021 5th CAA International Conference on Vehicular Control and Intelligence (CVCI).

[16] Šelek A., Hrabar I., Šteković S. Reactive navigation of the Ackermann steering robot in unknown environments. Proceedings of the 2025 11th International Conference on Automation, Robotics, and Applications (ICARA).

[17] Alhadad L.M., Akhmad H.R., Novel A., Widyatmaja M.D.S., Sembiring J., Loda K.B. Wearable universal long-range hand-gestured UAV radio control. Proceedings of the 2023 IEEE International Conference on Aerospace Electronics and Remote Sensing Technology (ICARES).

[18] Trinitatova D., Shevelo S., Tsetserukou D. Towards intuitive drone operation using a handheld motion controller. Proceedings of the 2025 20th ACM/IEEE International Conference on Human-Robot Interaction (HRI).

[19] ExpressLRS. High Performance Open Source Radio Control Link. Expresslrs.org. https://www.expresslrs.org/.

[20] Chen C.L., Huang S.H., Zhou J.H. Mobile robot localization by tracking built-in encoders. Proceedings of the 2014 International Symposium on Computer, Consumer and Control (IS3C).

[21] Sibai F.N., Trigui H., Zanini P.C., Al-Odail A.R. Evaluation of indoor mobile robot localization techniques. Proceedings of the 2012 International Conference on Computer Systems and Industrial Informatics (ICCSII).

[22] QuickPID: A Fast PID Controller with Multiple Options. Various Integral Anti-Windup, Proportional, Derivative and Timer Control Modes. GitHub. https://github.com/Dlloydev/QuickPID.

[23] Śmietańska J. Spiral Length Calculator. Omni Calculator. https://www.omnicalculator.com/math/spiral-length.

[24] Jocher G., Qiu J., Chaurasia A. Ultralytics YOLO. GitHub. https://github.com/ultralytics/ultralytics.

